# Diagnostic accuracy of coronary computed tomography angiography for the evaluation of obstructive coronary artery disease in patients referred for transcatheter aortic valve implantation: a systematic review and meta-analysis

**DOI:** 10.1007/s00330-022-08603-y

**Published:** 2022-02-22

**Authors:** Marco Gatti, Guglielmo Gallone, Vittoria Poggi, Francesco Bruno, Alessandro Serafini, Alessandro Depaoli, Ovidio De Filippo, Federico Conrotto, Fatemeh Darvizeh, Riccardo Faletti, Gaetano Maria De Ferrari, Paolo Fonio, Fabrizio D’Ascenzo

**Affiliations:** 1 Department of Surgical Sciences, Radiology Unit, University of Turin, Via Genova 3, 10126 Turin, Italy; 2 Division of Cardiology, Department of Medical Science, University of Turin, Turin, Italy; 3 School of Medicine, Vita-Salute San Raffaele University, 20121 Milan, Italy

**Keywords:** Coronary artery disease, Transcatheter aortic valve replacement, Computed tomography angiography, Aortic valve stenosis

## Abstract

**Objective:**

To evaluate the diagnostic accuracy of coronary computed tomography angiography (CCTA) for the evaluation of obstructive coronary artery disease (CAD) in patients referred for transcatheter aortic valve implantation (TAVI).

**Methods:**

EMBASE, PubMed/MEDLINE, and CENTRAL were searched for studies reporting accuracy of CCTA for the evaluation of obstructive CAD compared with invasive coronary angiography (ICA) as the reference standard. QUADAS-2 tool was used to assess the risk of bias. A bivariate random effects model was used to analyze, pool, and plot the diagnostic performance measurements across studies. Pooled sensitivity, specificity, positive ( + LR) and negative (−LR) likelihood ratio, diagnostic odds ratio (DOR), and hierarchical summary ROC curve (HSROC) were evaluated. Prospero registration number: CRD42021252527.

**Results:**

Fourteen studies (2533 patients) were included. In the intention-to-diagnose patient-level analysis, sensitivity and specificity for CCTA were 97% (95% CI: 94–98%) and 68% (95% CI: 56–68%), respectively, and + LR and −LR were 3.0 (95% CI: 2.1–4.3) and 0.05 (95% CI: 0.03 – 0.09), with DOR equal to 60 (95% CI: 30–121). The area under the HSROC curve was 0.96 (95% CI: 0.94–0.98). No significant difference in sensitivity was found between single-heartbeat and other CT scanners (96% (95% CI: 90 – 99%) vs. 97% (95% CI: 94–98%) respectively; *p* = 0.37), whereas the specificity of single-heartbeat scanners was higher (82% (95% CI: 66–92%) vs. 60% (95% CI: 46 – 72%) respectively; *p* < 0.0001). Routine CCTA in the pre-TAVI workup could save 41% (95% CI: 34 – 47%) of ICAs if a disease prevalence of 40% is assumed.

**Conclusions:**

CCTA proved an excellent diagnostic accuracy for assessing obstructive CAD in patients referred for TAVI; the use of single-heartbeat CT scanners can further improve these findings.

**Key Points:**

• *CCTA proved to have an excellent diagnostic accuracy for assessing obstructive CAD in patients referred for TAVI.*

• *Routine CCTA in the pre-TAVI workup could save more than 40% of ICAs.*

• *Single-heartbeat CT scanners had higher specificity than others in the assessment of obstructive CAD in patients referred for TAVI.*

**Supplementary Information:**

The online version contains supplementary material available at 10.1007/s00330-022-08603-y.

## Introduction

Transcatheter aortic valve implantation (TAVI) represents the gold standard for treatment of severe aortic stenosis in patients at high and intermediate surgical risk [1]. Candidates for TAVI are an elderly and frail population with a high prevalence (up to 60%) of coronary artery disease (CAD).

Current guidelines recommend pre-procedural screening for CAD before valvular intervention [1]. Computed tomography angiography (CTA) has become the standard imaging method for pre-procedural TAVI assessment because it can evaluate non-invasively both the aortic arch and the peripheral vessels [1, 2]. Coronary computed tomography angiography (CCTA) has been recommended as an initial test in patients with low clinical likelihood of CAD due to its very high negative predictive value in this population [2], but the clinical value of this approach in the TAVI setting is still under evaluation. Indeed, patients with severe aortic stenosis have a high pre-test probability of obstructive CAD and present specific technical challenges to CCTA interpretation, including extensive coronary calcifications, frequent abnormal cardiac rhythm, and contraindication to nitrate administration [1].

To the best of our knowledge, only two meta-analyses [3, 4] have evaluated the diagnostic accuracy of CCTA in patients with aortic stenosis referred for aortic valve replacement (surgical or transcatheter). In the last few years, technological advances in CT scanners have resulted in improved image quality, allowing accurate assessment of coronary anatomy even in difficult technical settings [5, 6]; this aspect was not assessed by the two abovementioned meta-analyses, both published in 2018. Moreover, indications to TAVI are progressively extending to low-surgical-risk patients with lower pre-test probability of CAD [7, 8].

In this framework, the incorporation of coronary artery assessment into pre-TAVI CT evaluation has the potential to reduce the need for unnecessary ICA and total amount of contrast medium applied, making pre-procedural evaluation safer and faster with reduced cost [9, 10].

The aim of this systematic review and meta-analysis is to provide an updated overview of the diagnostic accuracy of CCTA for the evaluation of obstructive CAD among patients referred for TAVI.

## Materials and methods

### Protocol and registration

This systematic review and meta-analysis was performed according to the Preferred Reporting Items for Systematic Reviews and Meta-Analysis (PRISMA) extension for Diagnostic Test Accuracy (DTA) Studies [11]. The protocol was prospectively registered in the PROSPERO International register of systematic reviews with the ID number CRD42021252527.

### Eligibility criteria

The primary aim of this study was to evaluate the diagnostic accuracy of CCTA for obstructive coronary stenosis among patients referred for TAVI. Studies reporting data on CCTA for the evaluation of obstructive CAD were deemed eligible if all the following inclusion criteria were respected: (1) CCTA performed with at least a 64-slice CT scanner; (2) ICA performed in all patients and used as the reference standard; (3) sensitivity and specificity were reported or assessed by the published data. Obstructive CAD was defined in the meta-analysis as a narrowing of the coronary lumen by more than 50% on CCTA and a lumen diameter reduction of more than 50% on ICA.

### Study endpoints

The primary endpoint was the patient-level accuracy of CCTA to identify obstructive CAD. For the purpose of this analysis, non-evaluable segments were considered positive based on an intention to diagnose approach [12]. Secondary analyses included the evaluation of the accuracy of CCTA for obstructive CAD at the patient level, excluding patients with non-evaluable segments, at the vessel and at the segment level, evaluating also coronary artery bypass grafting (CABG) and stented coronary artery segments. Sensitivity was analyzed according to the risk of bias and applicability. Subgroup analysis was performed based on CT scanner characteristics. We identified three main CT scanner subgroups: (1) whole-heart coverage CT scanner—scanners with extensive detector coverage on the z-axis (i.e., the 160-mm scanners); (2) high temporal resolution CT scanners (i.e., dual-source scanners); (3) single-heartbeat CT scanners—scanner capable of acquiring the entire heart volume in a single beat, including both whole-heart CT scanners and high temporal resolution scanners with a large number of detectors (e.g., Somatom Force, Siemens Healthineers). In particular, we conducted three subgroup analyses to determine the effect of these technical CT parameters on diagnostic accuracy: (1) whole-heart coverage CT scanner vs. other CT scanners; (2) high temporal resolution CT scanners (i.e., dual-source CTs) vs. other CT scanners, and (3) single-heartbeat CT scanners vs. other CT scanners.

### Search strategy

Excerpta Medica dataBASE (EMBASE), Medical Literature Analysis and Retrieval System Online (PubMed/MEDLINE), and Cochrane Central Register of Controlled Trials (CENTRAL) were searched up to May 1, 2021. The string used is reported in the Supplementary Material. The reference lists of selected articles were also searched manually to identify additional eligible studies.

### Data collection, data extraction, and risk of bias and applicability

Two researchers (F.B. and A.S.) independently searched for studies fulfilling the inclusion criteria in a two-stage process: first by using title and abstract of the papers and then the full text. The reasons for excluding studies in this second phase were recorded. The results from both searches were compared and the discrepancies were discussed. In some cases of disagreement, the decision was reached by consultation with a third researcher (G.G.). All selected articles were automatically downloaded, imported, and de-duplicated in Microsoft Excel (Microsoft).

General characteristics included total number of patients, age, sex, body mass index, cardiovascular risk factor (i.e., diabetes, hypercholesterolemia, smoking, history, hypertension), known CAD, previous percutaneous coronary intervention or CABG and atrial fibrillation. Moreover, main CT scanner characteristics were recorded: number of detector rows, dual-energy techniques, tube voltage tube current, contrast media concentration, contrast media volume, heart rate during acquisition, and mean dose-to-length product.

The quality assessment of diagnostic accuracy studies 2 (QUADAS-2) tool [13] was used to assess the risk of bias of included studies (reported in Supplementary Material).

### Statistical analysis

Two-by-two contingency tables were extracted from each study and used to calculate sensitivity, specificity, positive (+LR) and negative (−LR) likelihood ratio, and diagnostic odds ratio (DOR) with 95% confidence interval (CI) of CCTA for the detection of significant coronary artery stenosis (in general, + LR > 10 and −LR < 0.1 demonstrates a satisfactory diagnostic performance [14]). A bivariate random effects model was used to analyze, pool, and plot the diagnostic performance measurements across studies. Derived logit estimates of sensitivity, specificity, and respective variances were used to construct a hierarchical summary ROC curve (HSROC). Heterogeneity between studies was evaluated utilizing Cochran’s *Q* and Higgins *I*^2^ statistics. Deeks’ funnel plot was used to assess publication bias. The patient-level clinical accuracy of CCTA was evaluated using the likelihood ratios to calculate post-test probability based on Bayes’ theorem with the use of Fagan’s nomograms, Likelihood ratio scattergram, and probability modifying plot.

The analyses were performed with STATA (version 16.1, Stata Corp LP) using the MIDAS module [15] and MetaDTA (Diagnostic Test Accuracy Meta-Analysis v2.01) [16]. A *p* value of less than 0.05 was considered statistically significant.

## Results

### Literature search and study characteristics

**Fig. 1:**
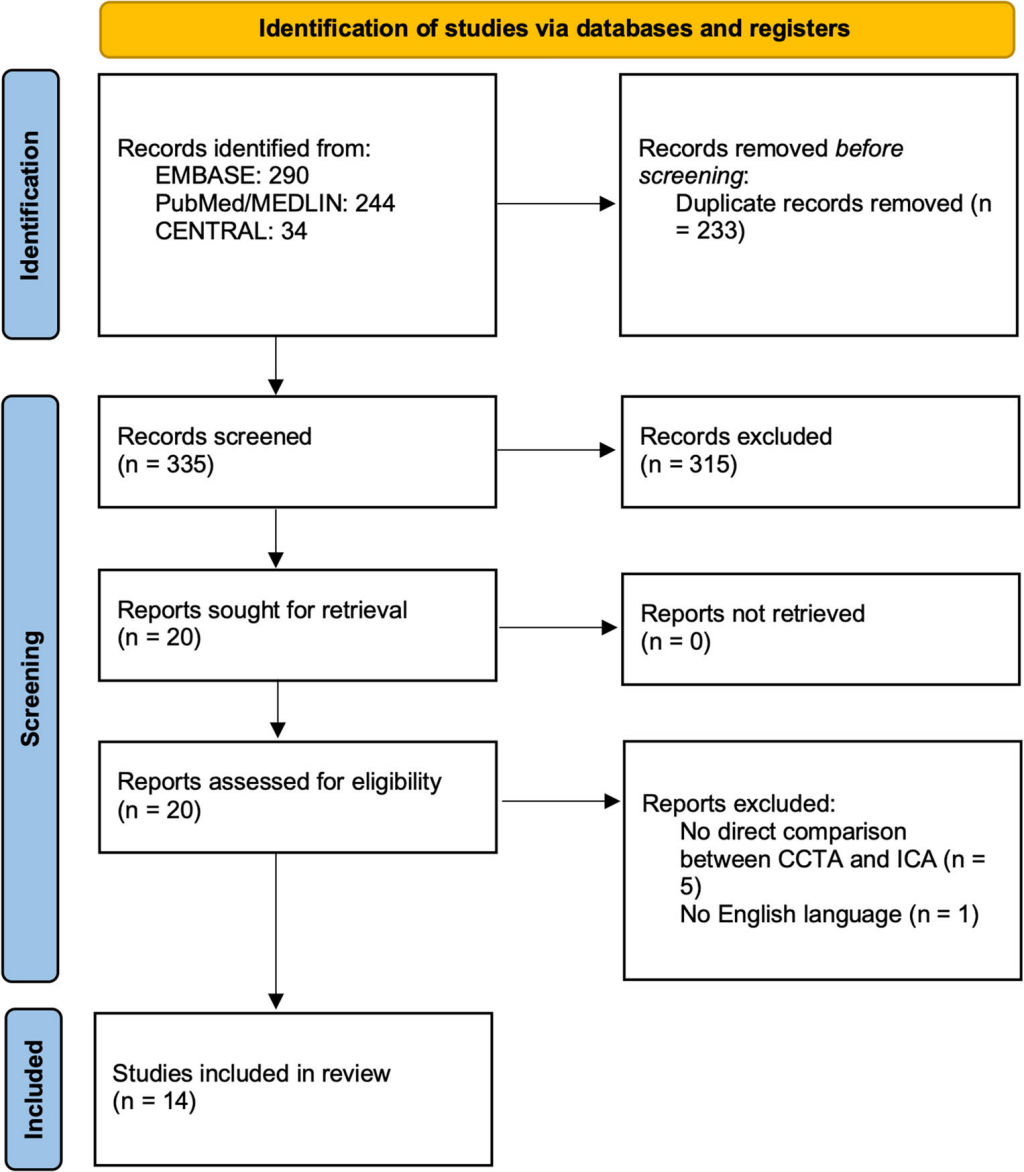
Flow diagram

The PRISMA 2020 flow diagram [17] for systematic reviews is reported in Figure 1. Fourteen studies with a total of 2533 patients were included in the analysis. Tables 1 and 2 report the baseline characteristic of the included patients and of the CT scanner used.

**Table 1:** Baseline characteristics of the study population

Author	Publication date	N	Age	Men	BMI	DM	HC	Smoke	AF	Sinus rhythm	HT	CAD	PCI	CABG
	Year	No.	Years	%	%	%	%	%	%	%	%	%	%	%
Pontone et al [26]	2011	60	80	36.6	25	13	40	25	0	100	67	37	24	16
Andreini [30]	2014	325	81.1	40.6	25.6	30	53	20	0	100	74	28	15	13
Hamdan et al [28]	2015	115	81.4	43.5	NR	30	70	36.5	7.8	92.2	85	52.2	29	20
Harris et al [12]	2015	100	79.6	61	NR	24	72	59	36	74	92	57	16	41
Opolsky et al [27]	2015	475	82	41	27.5	32	48	NR	19	75	95	67	48	19
Matsumoto et al [25]	2016	60	84.4	28.3	22.2	NR	NR	NR	NR	NR	NR	13	10	3.3
Rossi et al [19]	2017	140	82.3	48.6	27.1	21	59	19	0	100	75	0	0	0
Annoni et al [22]	2018	115	82.3	55.7	26.7	18	69	7	13	87	71	20.8	15	6.1
Hachulla [24]	2019	84	84.65	48.1	26.9	NR	NR	NR	NR	NR	NR	NR	NR	NR
Strong et al [20]	2019	200	83.4	40	26.6	28	74	21.5	34	76.5	93	0	0	0
Schicchi et al [29]	2020	223	79.2	NR	NR	NR	NR	NR	NR	NR	NR	51.6	35	16.6
Gohmann et al [21]	2020	388	79.6	50.8	29.2	13	59	8	NR	64.7	89	41.4	29	0
Shuai et al [23]	2020	121	73.3	47.1	22.6	26	12	25	27	73.8	37	NR	0	0
Meier et al [18]	2021	127	82.3	38.6	26.5	28	54	NR	NR	NR	77	38.6	17	0

*N* number, *BMI* body mass index, *DM* diabetes mellitus, *HC* hypercholesterolemia, *AF* atrial fibrillation, *HT* hypertension, *CAD* coronary artery disease, *PCI* percutaneous coronary intervention, *CABG* coronary artery bypass graft, *NR* not reported

**Table 2:** Baseline characteristics of the CT scanner used

Author	Publication date	CT scanner	X-ray source	Detector rows	Detector element *z*-dimension	Total detector *z*-axis coverage	Dual energy	rotation time	Intrinsic temporal resolution	Single-heart beat CT scanner	Tube voltage	Tube current
	Year		Detector design	Slices	mm	mm	In the present study	ms/rot	ms	Yes/no	Mean kVp	Mean mA
Pontone et al [26]	2011	LightSpeed VCT XT Scanner (GE Healthcare)	Single	64	0.625	40	No	350	175	No	120	650
Andreini [30]	2014	LightSpeed VCT XT Scanner (GE Healthcare)	Single	64	0.625	40	No	350	175	No	105	575
Hamdan et al [28]	2015	Brilliance iCT Elite (Philips Healthcare)	Single	128	0.625	80	No	270	135	No	100	485
Harris et al [12]	2015	Somatom Definition Flash Stellar (Siemens Healthcare)	Dual	64	0.6	38.4	No	285	75	No	NR	NR
Opolsky et al [27]	2015	Somatom Definition (Siemens Healthcare)	Dual	64	0.6	38.4	No	330	83	No	120	360
Matsumoto et al [25]	2016	Aquilion ONE Vision (Toshiba Medical Systems)	Single	320	0.5	160	No	275	137	Yes	100	382.5
Rossi et al [19]	2017	Somatom Definition Flash Stellar (Siemens Healthcare)	Dual	64	0.6	38.4	No	280	75	No	100	363.3
Annoni et al [22]	2018	Revolution CT (GE Healthcare)	Single	256	0.625	160	No	280	140	Yes	100	583.3
Hachulla [24]	2019	Somatom Definition Flash Stellar (Siemens Healthcare)	Dual	64	0.6	38.4	No	280	75	No	120	NR
Strong et al [20]	2019	Somatom Definition Flash Stellar (Siemens Healthcare)	Dual	64	0.6	38.4	No	280	75	No	110	NR
Schicchi et al [29]	2020	Somatom Force (Siemens Healthineers)	Dual	96	0.6	57.6	No	250	66	Yes	120	NR
Gohmann et al [21]	2020	Somatom Definition Flash Stellar (Siemens Healthcare)	Dual	64	0.6	38.4	No	280	75	No	86.7	NR
Shuai et al [23]	2020	Revolution CT (GE Healthcare)	Single	256	0.625	160	No	280	140	Yes	100	400
Meier et al [18]	2021	LightSpeed VCT XT; Revolution CT (GE Healthcare)	Single; single	64; 256	0.625	40; 160	No; no	350; 280	175; 140	No; yes	110	500

### Assessment of study quality

**Fig. 2:**
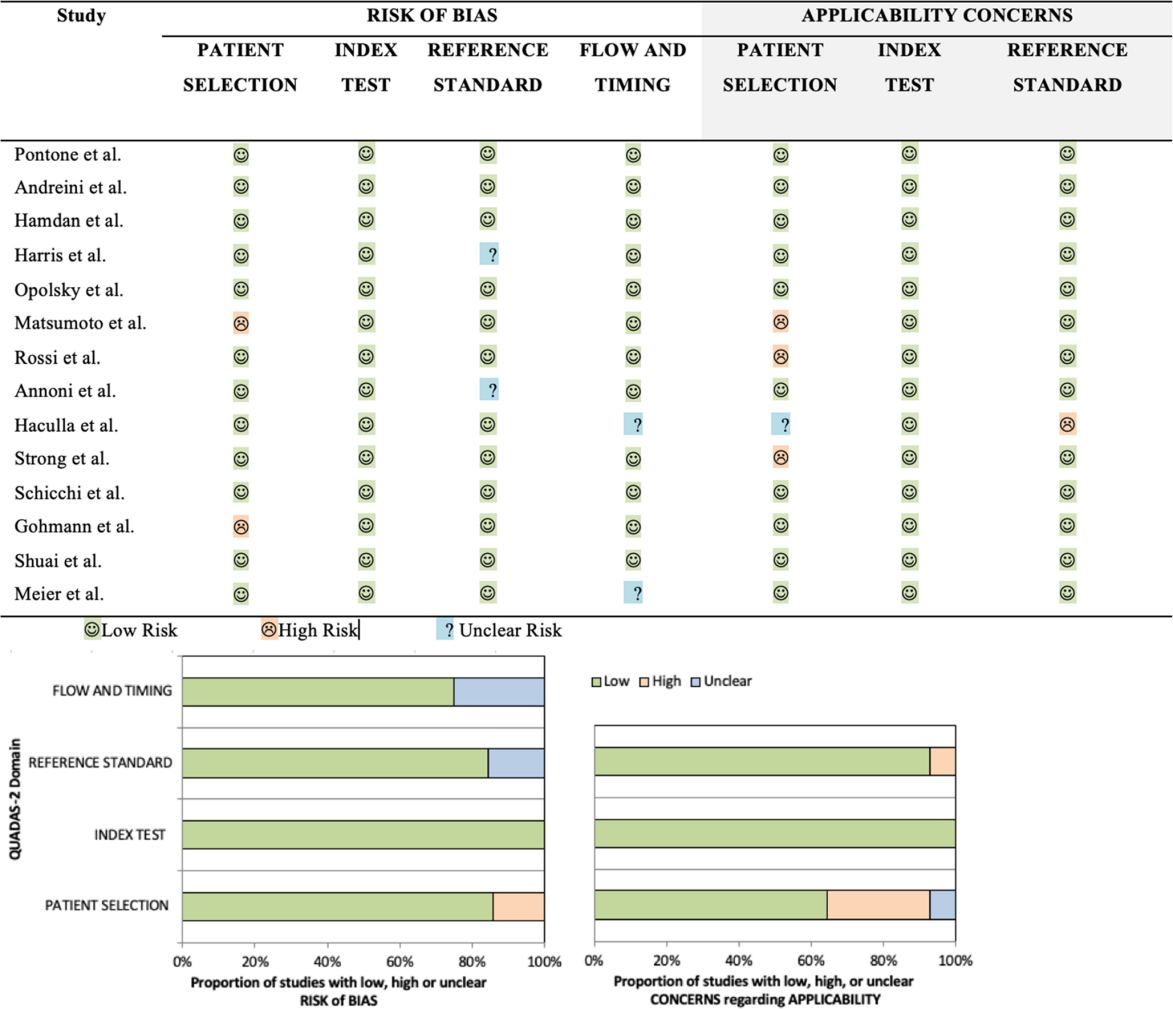
Quality assessment of diagnostic accuracy studies 2 (QUADAS-2) tool for risk of bias and applicability concern. Green represents low; yellow, high; and blue, unclear risk. On the top panel, QUADAS-2 was reported for each study and summarized in a bar graph on the bottom panel by stacked bars for each item

The QUADAS-2 Domain assessment is reported in Figure 2. The supplementary material contains the details of this analysis.

### Diagnostic accuracy: primary endpoint

**Fig. 3:**
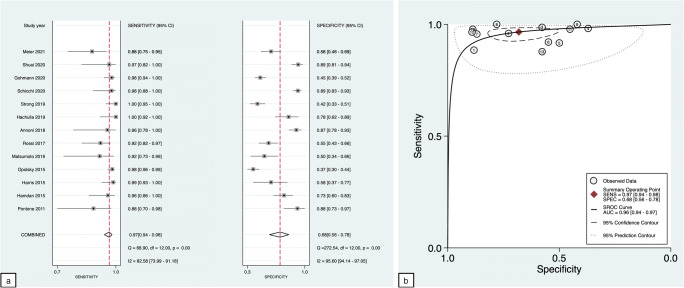
Summary forest plot is shown as paired plots, with sensitivity and specificity paired (**a**). HSROC plot at a patient-based level considering positive the nonvaluable segments with confidence and prediction regions around mean operating sensitivity and specificity point (**b**)

A total of 2228 patients were included in the analysis. For the purpose of this analysis, performed at the patient level, non-evaluable segments were considered positive based on an intention-to-diagnose approach. The pooled sensitivity and specificity for CCTA were 97% (94–98%) and 68% (56–68%), respectively, and the + LR and −LR were 3.0 (2.1–4.3) and 0.05 (0.03–0.09), with a DOR of 60 (30–121). The HSROC had an AUC = 0.96 (0.94–0.98). Table 3 shows sensitivity and specificity with % (95% CI) derived from each study included in the analysis. The summary forest plot and HSROC plot are reported in Figure 3.

**Table 3:** Summary sensitivities and specificities of CCTA for the identification of patients with obstructive coronary artery considering non-evaluable segments as positive

Author	Publication date	N	TP	FN	FP	TN	Se	Sp
	Year	No.	No.	No.	No.	No.	% (CI 95%)	% (CI 95%)
Pontone et al [26]	2011	60	23	3	4	30	88.5 (69.9 – 97.6)	88.2 (72.6 – 96.7)
Hamdan et al [28]	2015	115	47	2	18	48	95.9 (86.0 – 99.5)	72.7 (60.4 – 83.0)
Harris et al [12]	2015	100	73	1	11	15	98.7 (92.7 – 99.9)	57.7 (28.1 – 63.7)
Opolsky et al [27]	2015	475	265	5	129	76	98.2 (95.7 – 99.4)	37.1 (30.5 – 44.1)
Matsumoto et al [25]	2016	66	22	2	21	21	91.7 (73.0 – 99.0)	50.0 (34.2 – 65.8)
Rossi et al [19]	2017	145	58	5	37	45	92.1 (82.4 – 97.4)	54.9 (43.5 – 65.9)
Annoni et al [22]	2018	115	22	1	12	80	95.7 (78.1 – 99.9)	87.0 (78.3 – 93.1)
Strong et al [20]	2019	200	69	0	76	55	100.0 (94.8 – 100.0)	42.0 (33.4 – 50.9)
Schicchi et al [29]	2020	223	44	1	20	158	97.8 (88.2 – 99.9)	88.8 (61.9 – 82.9)
Gohmann et al [21]	2020	388	135	3	137	113	97.8 (93.8 – 99.6)	45.2 (32.9 – 51.6)
Shuai et al [23]	2020	130	28	1	11	90	96.6 (82.2 – 99.9)	89.1 (81.4 – 94.4)
Meier et al [18]	2021	127	43	6	33	45	87.8 (75.2 – 95.4)	57.7 (46.0 – 68.8)

*N* number of patients, *TP* true positive, *FP* false positive, *FN* false negative, *TN* true negative, *Se* sensitivity, *Sp* specificity

The per-patient analysis revealed a + LR of 3.03 (2.12–4.33) and a −LR of 0.05 (0.03–0.09) (i.e., with an estimated pre-test probability of CAD of 40%, a positive CCTA could increase the post-test probability to 67% and a negative CCTA can decrease the post-test probability to 3%, whereas in a hypothetical population with pre-test probability of 15%, the post-test probability can reduce to less than 1%).

**Fig. 4:**
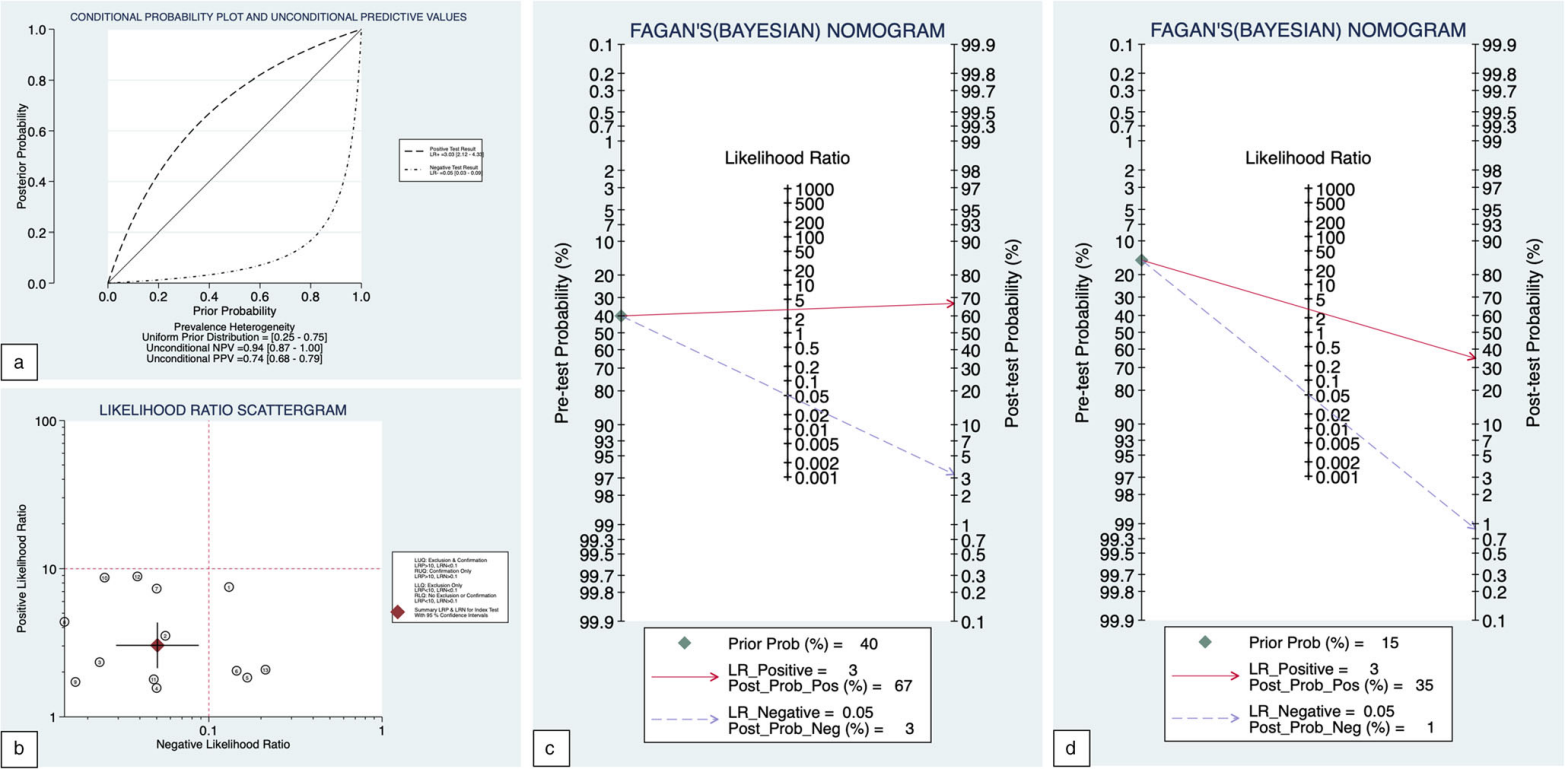
**a** The conditional probability modifying plot is a graphical sensitivity analysis of predictive value along a prevalence continuum designating low-risk to high-risk populations. It shows distinct curves for positive and negative testing. The user draws a vertical line from the chosen pre-test probability to the appropriate likelihood ratio line, then reads the post-test probability from the vertical scale. **b** The likelihood ratio scattergram represents the summary point of likelihood ratios calculated as functions of mean sensitivity and specificity. The summary point is located in the left lower quadrant: the CCTA has a likelihood ratio positive < 10 and a likelihood ratio negative < 0.1. Based on these considerations, the CCTA in patients referred for TAVI is useful for exclusion of CAD (when negative) rather than confirmation (when positive). **c**, **d** Fagan’s nomograms, with estimated pretest probability of 40% and 15%, respectively. A Fagan plot has a vertical axis on the left with the prior log-odds, a vertical axis in the middle with the log-likelihood ratio, and a vertical axis on the right with the posterior log-odds. The lines are then traced from the prior probability on the left to the likelihood ratios in the center, and then to the posterior probabilities on the right. Both plots highlight the strength of the CCTA in excluding the presence of CAD, with residual post-test probabilities of 3% and 1%, respectively

**Fig. 5:**
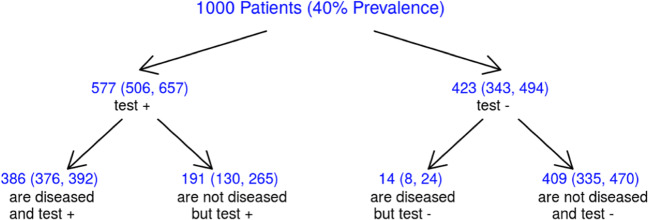
CCTA’s estimated impact in 1000 patients referred for TAVI. The numbers in brackets represent 95% confidence intervals

Fagan’s nomograms, with estimated pretest probability of 40% and 15%, Likelihood ratio scattergram and probability modifying plot are reported in Figure 4. In summary, estimating a disease prevalence of 40% in a population of 1000 patients, the study of coronary arteries with CCTA before the TAVI procedure would correctly avoid 409 (95% CI 335–470) ICAs (Figure 5).

Table 4 provides a summary of the CCTA diagnostic performance for the evaluation of obstructive CAD among patients referred for TAVI at a patient, vessel, and segment level. The secondary analysis is reported in the Supplementary Material.

**Table 4:** CCTA diagnostic performance for the evaluation of obstructive coronary artery disease in patients referred for TAVI

Analysis	*N*	TP	FP	FN	TN	Se	Sp	+ LR	− LR	DOR
	No.	No.	No.	No.	No.	% (CI 95%)	% (CI 95%)	% (CI 95%)	% (CI 95%)	*n* (CI 95%)
Patient level 1a)	2228	872	518	30	808	97 (94 – 98)	68 (56 – 78)	3.0 (2.1 – 4.3)	0.05 (0.03 – 0.09)	60 (30 – 121)
Patient level 1b)	794	252	123	16	403	94 (89 – 97)	80 (64 – 90)	4.6 (2.4 – 8.8)	0.08 (0.04 – 0.14)	59 (23 – 149)
Vessel level	6865	1307	1529	102	3927	92 (88 – 95)	79 (70 – 86)	4.4 (3.1 – 6.3)	0.10 (0.07 – 0.15)	42 (25 – 74)
Segment level	13525	1379	1408	81	10657	95 (89 – 98)	91 (83 – 95)	10.6 (5.6 – 20.4)	0.06 (0.03 – 0.12)	189 (61 – 583)

1a) Considering as positive the nonvaluable segments; 1b) including only patients with all segments evaluable

*N* number of cases included, *TP* true positive, *FP* false positive, *FN* false negative, *TN* true negative, *Se* sensitivity, *Sp* specificity, *+ LR* positive likelihood ratio, *− LR* negative likelihood ratio, *DOR* diagnostic odds ratio

### Sub-analysis: sensitivity and subgroup analysis

We found a high value for Cochran’s *Q* and *I*^2^, which indicates the presence of heterogeneity in the studies. As a result, we visually assessed the forest plot and HSROC and a significant heterogeneity in specificity was found, particularly in the forest plot (Figure 4a), where some studies fell outside the combined 95% CI.

For the purpose of the sub-analysis, performed at the patient level, non-evaluable segments were considered positive based on an intention-to-diagnose approach.

A sensitivity analysis including only five studies [23, 26–29] without high or unclear risk of bias or concerns regarding applicability showed similar results to the analysis containing all studies: a total of 1003 patients were included, the pooled sensitivity and specificity for CCTA were 96% (92 – 98%) and 79% (59 – 91%) respectively, and the + LR and −LR were 4.6 (2.2 – 9.7) and 0.05 (0.03 – 0.09), with a DOR of 94 (39–227). The HSROC had AUC = 0.97 (0.95 – 0.98).

The results of the subgroup analysis based the various CT scanner features are summarized in Table 5.

**Table 5:** CCTA diagnostic performance (comparison between single-heartbeat CT scanner vs. others) for the evaluation of obstructive coronary artery disease in patients referred for TAVI

CT scanner feature	Present	*N*	TP	FP	FN	TN	Se	Sp	+ LR	− LR	DOR	HSROC
		No.	No.	No.	No.	No.	% (CI 95%)	% (CI 95%)	% (CI 95%)	% (CI 95%)	*n* (CI 95%)	AUC
Whole-heart coverage CT scanner	Yes	311	72	44	4	191	95 (86 – 98)	80 (57 – 92)	4.6 (1.9 – 11.2)	0.06 (0.02 – 0.20)	73 (12 – 454)	0.96 (0.94 – 0.97)
	No	1790	757	441	20	572	97 (95 – 98)	65 (50 – 77)	2.8 (1.9 – 4.1)	0.04 (0.02 – 0.08)	64 (31 – 130)	0.97 (0.95 – 0.98)
High temporal resolution CT scanners	Yes	1615	687	419	15	494	98 (96 – 99)	59 (43 – 74)	2.4 (1.6 – 3.6)	0.03 (0.01 – 0.08)	73 (26 – 206)	0.97 (0.95 – 0.98)
	No	486	142	66	9	269	94 (89 – 97)	80 (66 – 89)	4.7 (2.6 – 8.5)	0.07 (0.04 – 0.14)	64 (23 – 179)	0.95 (0.92 – 0.96)
Single-heartbeat CT scanner	Yes	1567	713	421	19	414	96 (90 – 99)	82 (66 – 92)	5.4 (2.6 – 11.3)	0.05 (0.02 – 0.14)	112 (23 – 548)	0.97 (0.95 – 0.98)
	No	534	116	64	5	349	97 (94 – 98)	60 (46 – 72)	2.4 (1.7 – 3.3)	0.05 (0.03 – 0.09)	47 (25 – 91)	0.95 (0.93 – 0.97)

*N* number of cases included, *TP* true positive, *FP* false positive, *FN* false negative, *TN* true negative, *Se* sensitivity, *Sp* specificity, *+ LR* positive likelihood ratio, *− LR* negative likelihood ratio, *DOR* diagnostic odds ratio

In summary, the use of a whole-heart coverage CT increased specificity (*p* < 0.001) but did not affect sensitivity (*p* = 0.26); the use of high temporal resolution scanners increased sensitivity (*p* = 0.02) but decreased specificity (*p* < 0.001); and the use of single-heartbeat scanners increased specificity (*p* < 0.001) with no effect on sensitivity (*p* = 0.37).

**Fig. 6:**
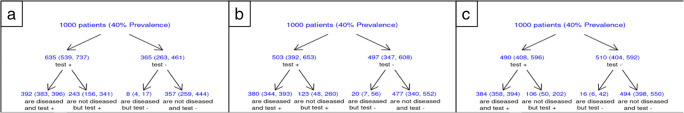
Impact of high temporal resolution CT scanners (i.e., dual-source CTs) (**a**), whole-heart coverage CT scanner (**b**), and single-heartbeat CT scanner (**c**) in 1000 patients referred for TAVI. The numbers in brackets represent 95% confidence intervals

To translate our findings into clinical practice, we estimated a disease prevalence of 40% in a 1000-patient population and evaluated coronary arteries with different CT scanners: a whole-heart coverage CT scanner could correctly avoid 477 (95% CI 340–552) ICAs, a high temporal resolution CT scanner could correctly avoid 357 (95% CI 259–444) ICAs, and a single-heartbeat CT scanner could correctly avoid 494 (95% CI 398–550) ICAs (Figure 6).

Furthermore, the percentage of non-evaluable patients with a whole-heart coverage CT scanner or a single-heartbeat CT scanner was 21.7% (13/60) compared to 37.1% (438/1180) with other CTs (*p* = 0.019). The percentage of non-evaluable patients using high temporal resolution CT scanners was 45.1% (368/815) compared to 19.5% (83/425) using other CTs (*p* = 0.001).

## Discussion

In this systematic review and meta-analysis, we investigated the diagnostic accuracy of CCTA for the assessment of obstructive CAD among patients referred for TAVI. Overall, CCTA prior to TAVI procedure provides high sensitivity (97%) with a good −LR (0.05).

This result highlights the effectiveness of CCTA to rule out significant CAD and to reduce unnecessary ICA procedures by 40.9%, aspiring its potential role as a gatekeeper test in this subgroup of patients. Furthermore, the use of new CT scanners, particularly single-heartbeat CT, has the potential to save the number of ICAs by up to 49.4%.

A recent meta-analysis on the same topic by van den Boogert et al [3] with included seven studies on a total of 1275 patients (all of these studies/patients were also incorporated into our meta-analysis) reported sensitivity, specificity, positive predictive value, and negative predictive value of 95.3% (93.3–96.9%), 65.3% (61.6–68.9%), 70.8% (68.6–72.9%), and 94.0% (91.6–95.8%) respectively. These results are very similar to our findings, despite the fact that the authors did not use a bivariate random effects model to summarize sensitivity, specificity, and their 95%, but rather a fixed effects model. In addition, they did not estimate +LR, −LR, and DOR.

Another meta-analysis on the performance of CCTA in patients with aortic stenosis undergoing surgery or transcatheter intervention by Chaikriangkrai et al [4] was performed. It included thirteen studies with a total of 1498 patients (6 of these studies, for a total of 1135 patients, were also incorporated into our meta-analysis); the results showed sensitivity = 95% (93–97%), specificity= 79% (68–86%), +LR = 4.48 (2.96–6.78), −LR = 0.06 (0.04–0.09) and AUC= 0.96 (0.95–0.98). The obtained specificity is slightly higher than our finding. This may be partially due to the population included in their study: in fact, in the subgroup analysis comparing patients who underwent surgery with those who underwent percutaneous intervention, the latter group showed a lower specificity (albeit not significant) (83% (77–87%) vs. 74% (51–88%)). This difference would be further increased by removing the study of Andreini et al [30] from their meta-analysis, thus increasing the specificity in the percutaneous intervention group even if it excluded non-evaluable patients. Another explanation could be the difference in disease prevalence between the two groups (48% in transcatheter vs. 29% in surgical): subjects who underwent transcatheter replacement were typically more fragile and at higher risk than those who underwent surgery, and this may increase the number of non-evaluable segments, thus reducing specificity.

In accordance with the European Society of Cardiology (ESC) guidelines [1], CCTA with its high negative predictive value is considered useful to exclude CAD in patients undergoing TAVI who are at low risk for atherosclerosis. This is in perfect agreement with our findings: a patient with a pre-test probability of 15% has a post-test probability of less than 1%, if the CCTA is negative.

However, our data seem to support a slightly broader usefulness and feasibility of CCTA even for patients with intermediate risk of CAD. In fact, on the basis of our findings, a patient with a pre-test probability of 40% (i.e., the prevalence of the disease in our population = 40%), in case of CCTA negativity, has a post-test probability of about 3%. This last finding is supported by a recent meta-analysis [31] of prospective studies comparing CCTA with coronary angiography as the reference standard. It highlighted the role of CCTA in patients with a low-to-intermediate pretest probability of CAD, emphasizing how this population could benefit the most from CCTA to rule in and rule out significant CAD.

The European Society of Cardiovascular Radiology (ESCR) consensus document [32] recently reported that CCTA should not be used routinely for pre-procedural assessment of CAD; however, they also added that, as technology evolves, CCTA can be used on a “case-by-case” basis, according to local expertise and available equipment and primarily to exclude significant coronary stenosis. This last statement was one of the starting points for our subgroup analyses. Technological advances in CT scanners were notable in recent years: the ideal CT scanner for cardiac imaging has high spatial and temporal resolution, covers the entire cardiac volume in a single rotation, and is ALARP (as low as reasonably practicable) compliant [33].

The abovementioned meta-analyses on a similar population [3, 4], published in 2018, did not analyze this aspect: (a) Regarding the high temporal resolution CT scanners, only three studies [12, 19, 27] analyzing 100, 475, and 145 patients, respectively, were included. (b) Concerning whole heart and single-heartbeat CT scanner, only one study [25] containing 66 patients was included.

In the period between those meta-analyses and our study, seven more papers using these technologies were published [18, 20–24, 29]. Therefore, we planned a sub-group analysis to explore the benefits of high intrinsic TR obtained by dual-source CT scanner, whole-heart coverage CT scanner, and single-heartbeat CT scanner. According to our findings, the use of CT scanners with high temporal resolution seems to improve sensitivity with lower specificity, whereas the use of whole-heart coverage and single-heartbeat CT scanners resulted in a higher specificity.

Single-heartbeat CT scanners in the evaluation of obstructive CAD prior to TAVI could correctly avoid up to 49.4% of ICAs. This data may be at least partially related to the increased number of segments, which allows accurate assessment of coronary arteries even in challenging population. The non-assessable segments were considered positive, leading to higher sensitivity and lower specificity in case of a high number of non-assessable segments. Our results confirm in a larger population the findings of Meier et al [18], who reported that the use of a 64-row scanner (compared to a 256-row scanner) for evaluating CAD in a pre-TAVI population was the only parameter in a multivariate analysis to be associated with a high risk of unanalyzable images. Our findings are consistent also with the meta-analysis of Haase et al [31], who reported that using a CT scanner with more than 64 detector rows led to higher sensitivity (93.4% vs 86.5%, *p* = 0.002) and specificity (84.4% vs 72.6%, *p* = 0.001) in ruling out or confirming CAD patients with a pretest probability of CAD ranging from 7 to 67%.

From a clinical point of view, it has been reported that patients with a large area of myocardium at ischemic risk may benefit the most from revascularization intervention prior to TAVI, and the proximal segment stenosis is prognostically more important than distal [34]. Unfortunately, the only study included in this meta-analysis that allows this type of analysis is that of Andreini et al [30], where the exclusion of non-assessable segments gives a boost to the specificity of CCTA, which appears superior even in the study of distal vs. proximal segments [35, 36].

Further studies are needed to evaluate the real impact of CCTA in clinical practice; a randomized controlled trial could be proposed to evaluate the prognostic impact of patients undergoing CCTA pre-TAVI vs. a group not undergoing CCTA; also, the actual potential of CCTA in the study of proximal segments in such a selected population remains to be explored.

This meta-analysis has some limitations. First of all, a relatively low number of studies met the selection criteria. Unfortunately, only a fraction of the studies reported the analyses at patient level, either by considering non-analyzable segments as positive or by excluding them, and therefore it was not possible to create 3 × 2 tables [37]. Moreover, despite relevance from a clinical point of view, we were not able to compare performance among high vs. low calcium and arrhythmic and vs. non-arrhythmic patients. Almost all studies included are retrospective cohorts; only one is prospective and none a randomized controlled study. In addition, many of the included studies are small in size. The studies’ overall quality was however adequate for analysis according to the QUADAS-2 evaluation, despite the presence of some unclear or high-risk items.

In conclusion, CCTA proved to have excellent diagnostic accuracy for assessing obstructive CAD in patients referred for TAVI. Routine CCTA assessment of coronary arteries as part of the pre-TAVI workup could save more than 40% of ICAs. The use of single-heartbeat CT scanners, which provide higher specificity, can further improve these findings.

## Supplementary Information

ESM 1 (DOCX 871 kb)

## Abbreviations

CAD: Coronary artery disease
CCTA: Coronary computed tomography angiography
CTA: Computed tomography angiography
DOR: Diagnostic odds ratio
HSROC: Hierarchical summary receiver operating characteristic curve
ICA: Invasive coronary angiography
−LR: Negative likelihood ratio
+ LR: Positive likelihood ratio
PCI: Percutaneous coronary intervention
PTP: Post-test probability
TAVI: Transcatheter aortic valve implantation

## Declarations

### Guarantor

The scientific guarantor of this publication is Prof. Paolo Fonio, chief of Diagnostic and Interventional Radiology at the University of Turin.

### Statistics and biometry

Dott. Marco Gatti, one of the authors, kindly provided statistical advice for this manuscript.

### Methodology

• Meta-analysis
