# Supplementary material for: Diagnostic accuracy of coronary computed tomography angiography for the evaluation of obstructive coronary artery disease in patients referred for transcatheter aortic valve implantation: a systematic review and meta-analysis
Source: Eur Radiol. 2022 Feb 22;32(8):5189–200. doi: 10.1007/s00330-022-08603-y (PMC9279258; doi:10.1007/s00330-022-08603-y)
Supplement: Supplementary file 1 — (DOCX 871 kb) [file 330_2022_8603_MOESM1_ESM.docx]

***SUPPLEMENTARY MATERIAL***

***Search strategy – string***

“((coronary computed tomography) OR (CCTA) OR (CT)) AND ((transcatheter aortic valve implantation) OR (transcatheter aortic valve replacement) OR (TAVI) OR (TAVR)) AND ((coronary artery disease) OR (CAD) OR (coronary stenosis) OR (coronary tree)) NOT (editorial) NOT (review) NOT (letter) NOT (case report) AND ('article' OR 'article in press')”.

***Assessment of Study Quality***

With regard to the risk of bias in patient selection, two studies were rated at high risk because one [18] did not report whether the enrolled patients were consecutive or not and the other study [19] divided the initial population into two groups: a) undergoing CCTA and ICA (388 patients included in the meta-analysis) and b) undergoing only CCTA (72 patients) on the basis of clinical decision (i.e. negative CCTA examination).

All studies were rated at low risk for the index test, whereas regarding the reference standard two [12, 20] were rated as unclear because they did not report whether the quantitative assessment of ICA was blinded to CCTA. Considering flow and timing, two studies [21, 22] did not report the interval between ICA and CCTA and hence were rated as unclear. With regard to concerns regarding applicability, four studies were rated high in patient selection [18, 23–25] because in their population there was a very low percentage of patients with CAD (below 20%, even 0% in two of them), while another study [21] was rated as unclear because it did not mention the number of patients with prior CAD, previous PCI or CABG. The Deeks’ funnel plot (**figure s1**) resulted in a p = 0.31, suggesting symmetry in the data and a low likelihood of publication bias. Furthermore, influence analysis revealed that all of the included studies fell below the red-dotted line, and outlier detection analysis revealed no outlier value, indicating that all 14 studies should be included (**figure s2)**.

***Diagnostic Accuracy: secondary analysis***

A total of 794 patients were included in the analysis at the patient-level including only patients with all segments evaluable. In this subgroup, 63.6% of patients were included (789 out of 1240). In particular, 0% of patients were excluded (0/140) in the study by Rossi et al. [23], 13% (8/60) in the study by Pontone et al. [26], 20% (62/243) in the study by Andreini et al. [27], 22% (13/60) in the study by Matsumoto et al. [18] and up to 55% (259/475 and 109/200) in the study by Opolsky et al. [28] and Strong et al. [24]. The pooled sensitivity and specificity for CCTA were 94% (89–97%) and 80% (64–90%) respectively, and the +LR and -LR were 4.6 (2.4–8.8) and 0.08 (0.04–0.14), with a DOR of 59 (23–149). The HSROC had an AUC = 0.95 (0.92–0.96). The summary forest plot and HSROC plot are reported in **Figure s3.**

A total of 6865 vessels were included in the analysis at the vessel-level. The pooled sensitivity and specificity for CCTA were 92% (88–95%) and 79% (70–86%) respectively, and the +LR and -LR were 4.4 (3.1–6.3) and 0.10 (0.07–0.15), with a DOR of 42 (25-74). The HSROC had an AUC = 0.94 (0.91-0.95). The summary forest plot and HSROC plot are illustrated in **Figure s4.**

A total of 13525 coronary artery segments were included in the analysis at the segment-level. The pooled sensitivity and specificity for CCTA were 95% (89–98%) and 91% (83–95%) respectively and the +LR and -LR were 10.6 (5.6–20.4) and 0.06 (0.03-0.12), with a DOR of 189 (61–583). The HSROC had an AUC = 0.98 (0.96–0.99). The summary forest plot and HSROC plot are shown in **Figure s5.**

Comparison between the diagnostic performance of CCTA in proximal and distal coronary artery segments was not possible. Only one paper [27] showed such an analysis (excluding non-assessable segments in the analysis) in proximal vs. distal segments: sensitivity of 94.7% (86.9-98.5%) vs. 88.5% (81.1-93.7%), p = 0.20 and specificity of 98.6% (97.7-99.2%) vs. 99.4 (99.1-99.7%), p = 0.01; PPV of 81.6% (73.1-87.9) vs. 84.8% (77.7-89.9), p = 0.57 and NPV of 99.7% (99.1-99.9) vs. 99.6% (99.3.99.8), p > 0.99.

A total of 791 CABG were sub-analyzed. The pooled sensitivity and specificity for CCTA were 96% (92–98%) and 99% (93–100%) respectively, and the +LR and -LR were 69.0 (13.5–352.8) and 0.04 (0.02–0.08) with a DOR of 1735 (287–10490). A total of 62 stented segments were sub-analyzed. Pooled sensitivity and specificity for CCTA were 92% (64–99%) and 82% (68–91%) respectively and the +LR and -LR were 5.03 (2.73–9.26) and 0.09 (0.01–0.62).

**FIGURE LEGENDS**

**Figure s1**. Deeks’ funnel plot with superimposed regression line. The inverse of the square root of the effective sample size is displayed on the vertical axis. The diagnostic odds ratio is shown on the horizontal axis. p=0.31 reflects a symmetrical funnel shape and the absence of publication bias.


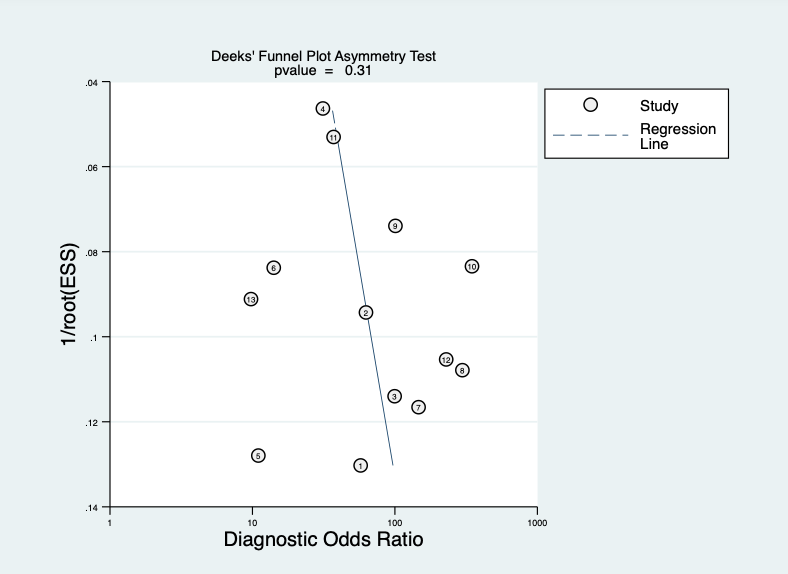


**Figure s2**. Composite graphic of model diagnostic: quantile plot of residual based goodness-of fit (panel a); Chi-squared probability plot of squared Mahalanobis distances for assessment of the bivariate normality assumption (panel b); spikeplot for checking for particularly influential observations using Cook’s distance (panel c) and a scatter plot for checking for outliers using standardized predicted random effects (panel d).


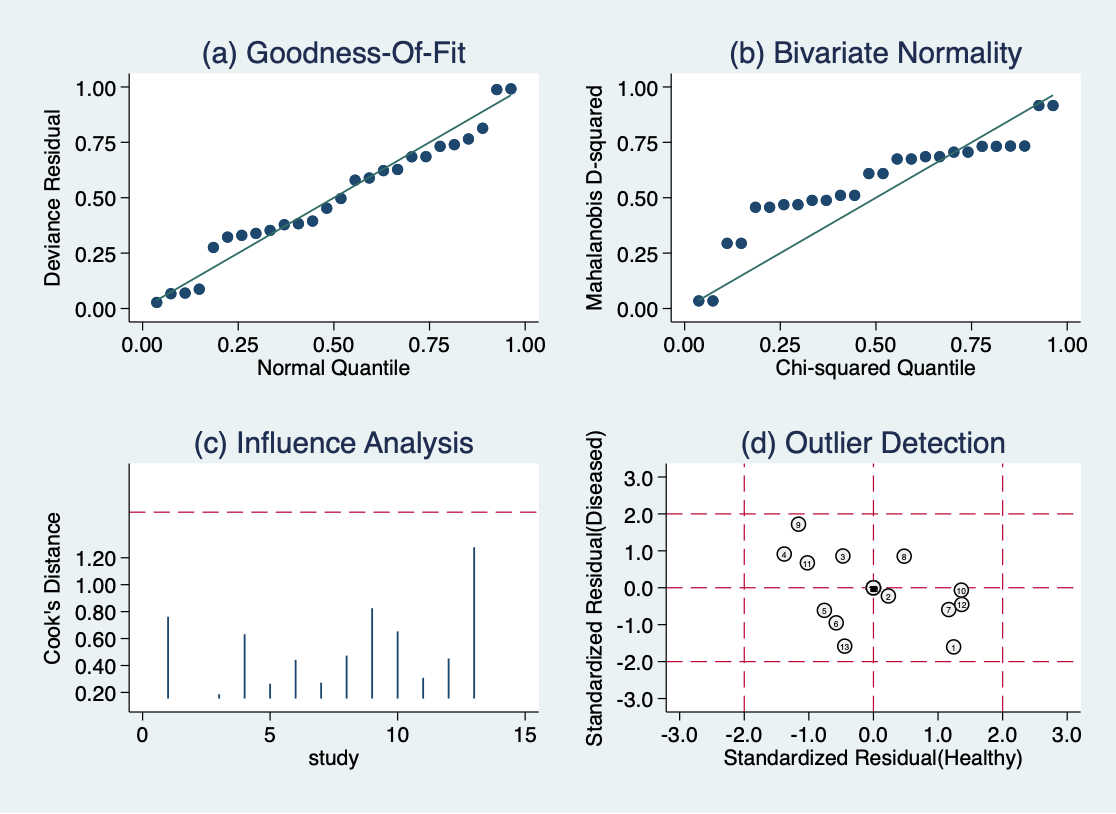


**Figure s3.** Summary forest plot and HSROC plot at a patient-level including only patients with all segments evaluable.


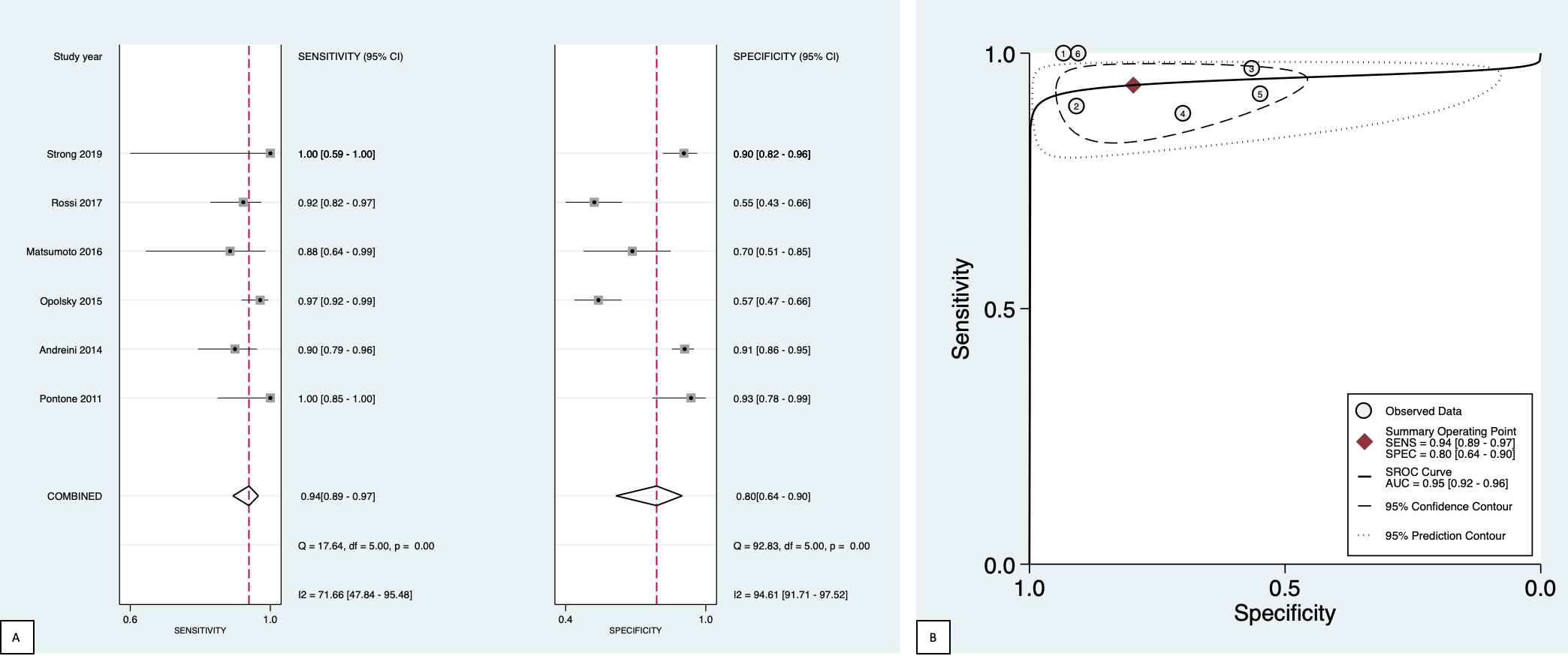


**Figure s4.** Summary forest plot and HSROC plot at the vessel-level.


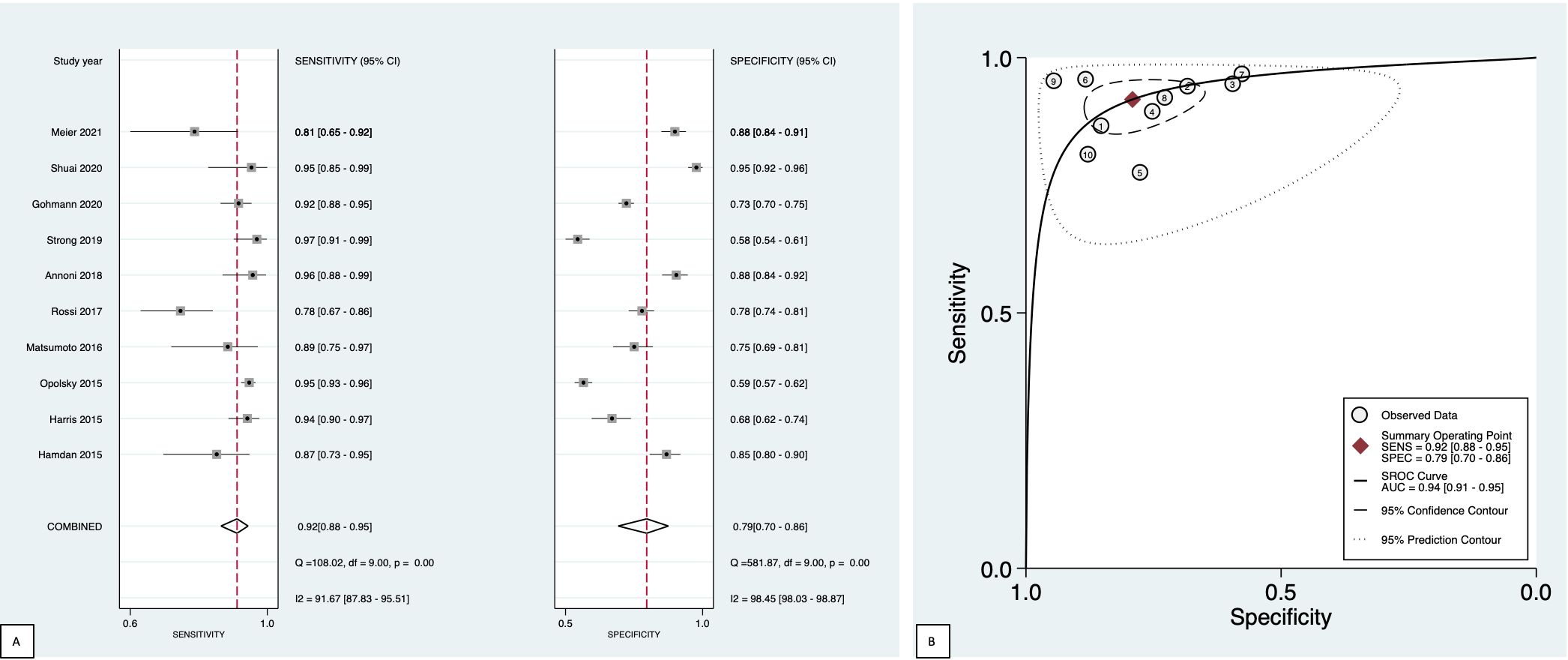


**Figure s5.** Summary forest plot and HSROC plot at the segment-level.

**
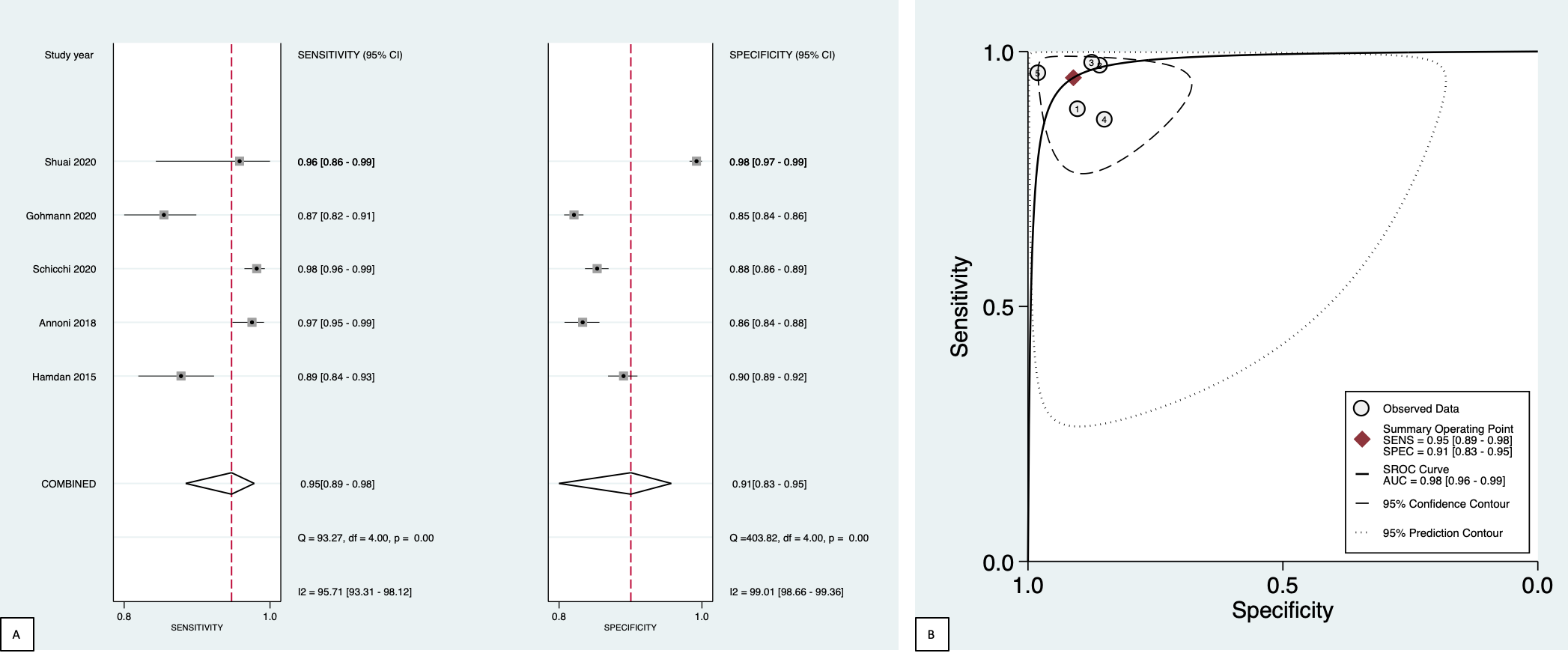
**
